# Biodistribution Profile of Magnetic Nanoparticles in Cirrhosis-Associated Hepatocarcinogenesis in Rats by AC Biosusceptometry

**DOI:** 10.3390/pharmaceutics14091907

**Published:** 2022-09-08

**Authors:** Guilherme A. Soares, Gabriele M. Pereira, Guilherme R. Romualdo, Gabriel G. A. Biasotti, Erick G. Stoppa, Andris F. Bakuzis, Oswaldo Baffa, Luis F. Barbisan, Jose R. A. Miranda

**Affiliations:** 1 Department of Biophysics and Pharmacology, Institute of Biosciences, São Paulo State University—UNESP, Botucatu 18618-689, SP, Brazil; 2 Department of Pathology, Botucatu Medical School, São Paulo State University (UNESP), Botucatu 18618-689, SP, Brazil; 3 Department of Strucutral and Functional Biology, Institute of Biosciences, São Paulo State University—UNESP, Botucatu 18618-689, SP, Brazil; 4 Institute of Physics, Federal University of Goiás, Goiânia 74690-900, GO, Brazil; 5 Faculty of Philosophy, Sciences and Letters at Ribeirão Preto, University of São Paulo, Ribeirão Preto 14040-900, SP, Brazil

**Keywords:** AC biosuceptometry, magnetic nanoparticles, cirrhosis-associated rat hepatocarcinogenesis, nanotechnology

## Abstract

Since magnetic nanoparticles (MNPs) have been used as multifunctional probes to diagnose and treat liver diseases in recent years, this study aimed to assess how the condition of cirrhosis-associated hepatocarcinogenesis alters the biodistribution of hepatic MNPs. Using a real-time image acquisition approach, the distribution profile of MNPs after intravenous administration was monitored using an AC biosusceptometry (ACB) assay. We assessed the biodistribution profile based on the ACB images obtained through selected regions of interest (ROIs) in the heart and liver position according to the anatomical references previously selected. The signals obtained allowed for the quantification of pharmacokinetic parameters, indicating that the uptake of hepatic MNPs is compromised during liver cirrhosis, since scar tissue reduces blood flow through the liver and slows its processing function. Since liver monocytes/macrophages remained constant during the cirrhotic stage, the increased intrahepatic vascular resistance associated with impaired hepatic sinusoidal circulation was considered the potential reason for the change in the distribution of MNPs.

## 1. Introduction

The liver is a solid organ that is divided into two portions: (1) a parenchymal portion, which is composed of hepatocytes and biliary cells, and (2) a non-parenchymal portion, constituted by Kupffer cells (KCs), sinusoidal endothelial cells (LSECs), and resting hepatic stellate cells (HSCs) [1]. The KCs are resident macrophages that specialize in phagocytosis and cytokine release, acting as the liver’s first immune defense [2]. KCs are associated with the LSECs that line the hepatic sinusoids. The HSCs are spread throughout the Disse space and are responsible for storing vitamin A and secreting limited amounts of extracellular matrix (ECM) proteins under physiological conditions [3].

The liver, under homeostasis, displays an extensive range of functions, such as the regulation of blood volume and immunity, drug detoxification, endocrine control of growth, lipid and cholesterol homeostasis, and the metabolism of nutrients. It also features a regenerative capacity through hepatocytes [4,5,6] Nonetheless, this organ may develop several chronic diseases, including non-neoplastic and neoplastic diseases. Hepatocellular carcinoma (HCC), the main primary hepatic malignancy, stands out for its current epidemiological burden, as it ranks fourth among the most common cancers and it is the sixth most common cause of cancer-related deaths worldwide [7]. HCC usually emerges in the context of hepatic fibrosis/cirrhosis (70–95% of cases) [8] and also features a poor prognosis, with a median survival time of 11 months and survival rates of 19 to 29% at 3 years after diagnosis [9,10]. Furthermore, a 53% to 60% growth in both the incidence of and mortality from HCC is estimated over the next 20 years [7]. Such epidemiological data elicit the need for new diagnostic, preventative, and therapeutic tools for this malignance.

Under the known risk conditions—i.e., chronic hepatitis B and C infections, non-alcoholic liver disease, and alcohol intake—HCC gradually emerges in the context of tumor-promoting inflammation/hepatocyte injury hallmarks, HSC activation, and HSC sand macrophage pro-inflammatory crosstalk, culminating on collagen production. Collagen progressively accumulates, leading to liver fibrosis, and the end stage of this process is called cirrhosis, which is characterized by a marked impairment of liver function and an increased risk for HCC development [11]. In order to investigate the different aspects of cirrhosis-associated hepatocarcinogenesis, experimental models, including chemically induced models, have been widely applied in translational research [11,12,13,14]. These models use chemical hepatotoxins that induce (pre)neoplastic lesions in a cirrhotic background, as in the diethylnitrosamine (DEN)-initiated and thioacetamide (TAA)-promoted model [15]. These murine models gather morphological and molecular similarities to the corresponding human disease, enabling translational research on hepatocarcinogenesis [11,13] and nanotechnology studies. 

Despite liver fibrosis not having clear symptoms, its early detection is essential for preventing the further aggravation to other diseases such as cirrhosis and HCC and for providing beneficial future treatments [14,15]. Although a percutaneous liver biopsy is an invasive strategy and presents several drawbacks, such as sampling error and cost, this diagnostic procedure is usually always associated with non-invasive diagnostic methods and serum biochemistry [16,17,18]. In addition to non-invasive staging of hepatic fibrosis using magnetic resonance imaging (MRI) and computed tomography (CT), ultrasonography is a widely used accurate diagnostic imaging tool [19,20,21,22]. This diagnostic method is also inexpensive, supporting its practical use. Nevertheless, these imaging methods have drawbacks that make detecting fibrosis and cirrhosis at early stages difficult, and there are also drawbacks related to the experience level of the operator. Furthermore, these methods are not indicated for obese patients [18,23]. Despite a routine MRI examination presenting advantages such as its ability to reach deep tissue in the liver with a high spatial resolution, which allows for a complete characterization of liver disease processes, it has some limitations [14]. At the same time, the disadvantages of CTs are the need for ionizing radiation and the existence of respiratory motion artifacts.

Several conventional approaches have been employed to suppress hepatic inflammation/scar deposition using antifibrotic drugs to treat liver diseases. However, most of these conventional therapies are ineffective because the drug delivery is not specific, since specific hepatic cell types are responsible for hepatic inflammation/fibrosis [4,24,25]. In this way, the difficulty of delivering a sufficient dose of pharmacological agents to the liver is associated with the non-specificity of targeting cellular structures, indicating that treating liver diseases remains a challenge.

Nanomaterials have attracted attention in the development of nanotechnology due to the possibility of their use as multifunction probes for diagnoses and treatments in recent years [26,27,28,29,30,31]. Nanoparticles have great potential for several biomedical applications since they have interesting properties, such as a reduced size, shape manipulation, and the possibility of conjugation with other materials and molecules. A class of nanoparticles that has several advantages due to the intrinsic properties and biocompatibility of its members is magnetic nanoparticles (MNPs). Over the last few years, MNPs have been used in many theranostic applications [32,33,34,35], including diagnosing and treating hepatic diseases [32,33,34]. The magnetic nanoparticle-based diagnosis and treatment of liver diseases has shown great potential, since MNPs present advantages such as (i) easy functionalization and conjugation with molecules and surface markers, which allows for targeting drug delivery agents to specific cell-type agents [35]; (ii) their ability to act as magnetic vectors to specific liver locations, since they respond strongly to an external magnetic gradient [36]; and (iii) their ability to act as labeling and tracking agents in imaging modalities, thus enhancing non-invasive approaches for investigating liver fibrosis conditions [37,38]. Within the parameters that determine the blood clearance pharmacokinetics of MNPs, the hydrodynamic size of MNPs is one of the most critical parameters that affect their biodistribution kinetics and uptake by the mononuclear phagocyte system (MPS) [39,40]. The MPS, which comprises dendritic cells, blood monocytes, and resident-tissue macrophages in several organs, is a specialized and selective structure that takes up nanoparticles in general. Usually, it has been reported through consistent evidence that nanoparticles presenting with hydrodynamic sizes within 15–100 nm are captured mainly by the liver and the spleen [41,42,43]. In comparison, nanomaterials smaller than 10 nm are likely to be eliminated through renal clearance [44,45,46].

Once administered intravenously, MNPs are substantially captured and retained in the liver, depending on physical factors such as coating, dose, and size [47]. The presence of fenestrated vasculature (sinusoids) and many Kupffer cells supports the significant amount of MNPs in the liver. Literature reports have indicated that the liver takes up around 30–99% of the MNPs in a dose [48,49,50,51]. Therefore, the high abundance of MNPs in the liver after intravenous injection and their superparamagnetic properties increase the potential of these materials to be used as a contrast agent to enhance the signal-to-noise ratio, which makes magnetic imaging modalities feasible for diagnosing liver diseases [52,53,54,55].

Over the years, several methods have been used to detect MNPs in tissues. These methods are classified into direct and indirect methodologies. For in vivo studies, MRI and magnetic particle imaging (MPI) are techniques that detect and visualize particles by their inherent properties and can contribute to determining the pharmacokinetics and biodistribution of MNPs [56]. 

Despite MRI being a consolidated methodology for imaging, in general, it involves a high cost and also has drawbacks regarding the differentiation of the position of MNPs with a low signal [7].

MPI emerged as an alternative and promising technique for MNP detection. The technique is based on the nonlinear magnetic response of the IONPs to an applied AC magnetic field and presents no depth limitation when used to directly measure the MNP concentration. However, MPI presents limitations regarding the complexity and associated high cost, so it is not widely used [57]. Nanoparticles can be detected through their conjugation to contrast agents or radioactive markers by using imaging methodologies such as near-infrared (NIR) fluorescence, positron emission tomography (PET), and single-photon emission computed tomography (SPECT) [58,59,60]. 

Electron paramagnetic resonance (EPR) and a superconducting quantum interference device (SQUID) are magnetometry techniques that are able to carry out ex vivo assessments. 

Within ex vivo methodologies, elemental analysis methodologies, such as inductively coupled plasma-atomic emission spectroscopy (ICP-AES) and Prussian blue analysis, show limitations in quantifying the exclusive iron from MNPs [61,62].

An alternate current biosusceptometry (ACB) system has been employed in biological applications involving MNPs because of its unique advantages, such as a low-cost versatility and a lack of specialized equipment required. The system also does not use ionization radiation and works in unshielded magnetic environments [63,64,65,66,67]. Recently, the system has been improved through a comprehensive mathematical and computational approach to quantitatively reconstruct 2D distributions of MNPs [68].

In a previous study, we undertook a pharmacological approach to understand hepatic MNP uptake through ACB imaging. However, the study was limited to quantifying the MNP distribution, potentially minimizing future pre-clinical applications. 

We emphasize that the paper presented here represents a significant improvement to the ACB system, mainly regarding MNP quantification in real time using high-quality quantitative images through the inverse problem solution. In addition, this work describes the use of a new MC-ACB system with a higher temporal resolution due to the number and density of detector coils and an additional biodistribution analysis.

Therefore, we decided to implement the MC-ACB system associated with MNPs to investigate a chronic liver disease that significantly impacts morbidity and mortality worldwide.

## 2. Materials and Methods

### 2.1. Magnetic Nanoparticles

Solutions of iron (III) chloride hexahydrate (FeCl_3_—purity 97–100%), manganese (II) chloride tetrahydrate (MnCl_2_ 4H_2_O—purity 98–100%), iron nitrate (Fe (NO_3_)_3_—purity 98–100%), and methylamine (CH_3_NH_2_—purity 99.5%) were purchased from Sigma-Aldrich (St. Louis, MO, USA). Acetone (purity 99.6%) and sodium citrate (Na_3_C_6_H_5_O_7_—purity 99–100%) were purchased from Cromoline, Diadema, Brazil.

We used citrate-coated manganese ferrite nanoparticles (Cit-MnFe_2_O_4_) synthesized by co-precipitation as described before [69,70]. Dynamic light scattering (DLS, Zetasizer NanoS Malvern Instruments, Malvern, UK) measurements showed the hydrodynamic diameter of the particles and zeta potential. Through a Jeol transmission electron microscope, model JEM 2100 (Tokyo, Japan), operating at 200 kV, we obtained images of the core diameter distribution of the MNPs. The magnetization curve of the Cit-MnFe_2_O_4_ MNPs was obtained using an ADE Vibrating Sample Magnetometer (VSM), model EV9 (MicroSense, EastLowell, MA, USA). The Cit-MnFe_2_O_4_ composition was assessed using an energy-dispersive X-ray spectroscopy (EDS) detector (Jeol, JSM-6610).

The X-ray diffraction patterns of the MNP powders were analyzed using a Shimadzu 6000 diffractometer (Shimadzu Corporation, Kyoto, Japan) in order to study the structural parameters of the MNPs. To ensure the success of the coating and confirm the presence of the magnetic nanoparticles, Fourier-transform infrared (FTIR) analysis was carried out using Varian IR 640 equipment.

### 2.2. Alternate Current Biosusceptometry

The system was a magnetic material detector working as a double magnetic flux transformer, and was composed of 19 drive and pickup coils. Both pairs were arranged on a first-order gradiometer to provide a good signal-to-noise ratio while reducing environmental noise and leading to the cancelation of the common mode. When the magnetic sample was near the pickup coils, the magnetic flux balance was altered, inducing an electric current in the pickup coils proportional to the volume δv and magnetic susceptibility χ. 

This signal was acquired using the same low-noise lock-in amplifier that recorded the excitation frequency components (10 kHz). After converting into a direct current signal (DC), the ACB signal was digitalized in real time using a National Instruments A/D board (20 Hz of the sampling rate).

The ACB signal intensity detected by the pickup coils depended on intrinsic coil parameters, such as the area of the detection coil, the number of turns, the magnetic flux change rate, and the amount of magnetic material. Detailed information can be found in [68].

**Figure 1:**
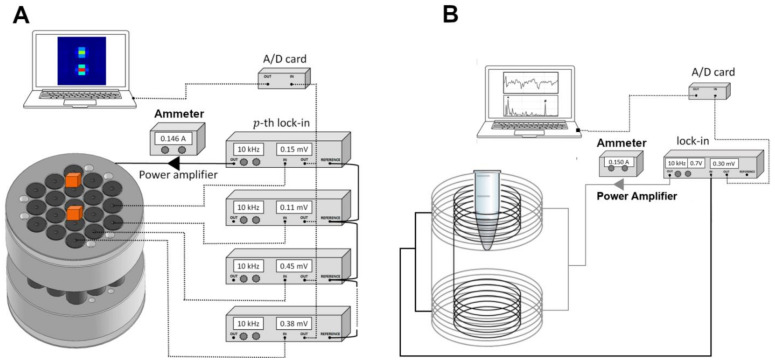
Schematic representation of both ABC setups utilized. (**A**) MC-ACB and (**B**) cavity ACB sensor.

We used two ACB setups for our measurements in this present study. The multichannel ACB system (MC-ACB) was employed to acquire the real-time biodistribution of MNPs simultaneously in blood circulation and the liver. Then, we utilized a suitable ACB sensor to quantify the final mass accumulated in each organ collected after the animal’s death [43]. Figure 1 presents the two ACB setups used in this work.

### 2.3. Experimental Design of Cirrhosis-Associated Hepatocarcinogenesis Model

The current cirrhosis-associated hepatocarcinogenesis model was based on a previously published protocol [11]. In this rodent study, 36 males (*Rattus norvegicus albinus*, Wistar, weighing 250–300 g) provided by the UNESP animal facility (São Paulo State University) were divided into two groups. The animals were maintained under suitable conditions at 21 °C ± 1 °C with a 12 h/12 h light/dark cycle, constant air filtration, and ad libitum feeding. All animal experiments were previously approved and performed following the recommendations issued by the National Council for Control of Animal Experimentation (CONCEA) and were approved by the Ethics Committee on Animal Use of the São Paulo State University (IBB/UNESP) under protocol 7571041120.

The animals were randomly assigned to one of two groups, of which one was subjected to a NaCl 0.9% solution treatment (SAL-control group) and the other was subjected to the chemically-induced cirrhosis-associated hepatocarcinogenesis model (DEN/TAA group) [11]. 

The animals received a single intraperitoneal injection of diethylnitrosamine (DEN, 200 mg/kg in 0.9% saline solution) (Sigma-Aldrich, USA) to initiate liver carcinogenesis. After two weeks, we assigned the animals to three cycles of thioacetamide (TAA) (200 mg/kg in 0.9% saline solution) (Sigma-Aldrich, USA). During the fibrosis/cirrhosis induction, each TAA cycle was achieved after two intraperitoneal injections (twice a week), with an interval of one week without receiving treatment. The model of cirrhosis/hepatocarcinogenesis was carried out for eight weeks.

The animals were subjected to femoral vein cannulation surgery for the intravenous administration of MNPs (dose of 32 mg/kg) under anesthesia (99% urethane—1.5 mg/kg.) Then, the animals were positioned on the MC-ACB detection coils to carry out the magnetic in vivo biodistribution monitoring (further described in Section 2.4). 

### 2.4. In Vivo Quantitative Imaging and Data Processing

We carried out quantitative MNP reconstruction by employing the MC-ACB, for which we had recently demonstrated the mathematical and computational approaches for improving the system’s ability to acquire quantitative information [68]. We monitored the MNP biodistribution and recorded heart and liver signals using the MC-ACB system. We reconstructed the MNP biodistribution from the quantitative real-time signals, represented in sequential images (frames) at a sampling frequency of 20 Hz. Regions of interest (ROIs) were selected for the frames corresponding to the signals from the organs of interest (liver and heart). We quantified a series of pharmacokinetic parameters from the MNP distribution signals of both organs over time.

### 2.5. Histopathological and Immunohistochemical Analysis

Liver tissue samples were washed, fixed in formalin, embedded in paraffin, and sequentially sectioned into 5 µm sections. Slides were stained with hematoxylin and eosin (H&E) and Sirius red (collagen deposition) for microscopic analysis. Other sections obtained on silane-coated slides (Starfrost, Lowestoft, UK) were subjected to immunohistochemical reactions to evaluate the expression of placental glutathione-S-transferase (GSTP) (i.e., preneoplastic and neoplastic marker) and CD68 (monocyte, macrophage, and Kupffer cell marker) antigens, as previously described [11,12]. 

The placental GST-P (π isoform) is expressed in initiated hepatocytes, but not in normal or non-initiated hepatocytes, indicating their role in hepatocarcinogenesis [71]. CD68 is a glycosylated type I transmembrane glycoprotein that is considered an important cytochemical marker for macrophages, especially in the histochemical analysis of inflamed tissues [72,73]. 

We performed a morphometric analysis (lesions and nodules positive for GST-P and collagen content) using the Leica Q-win Software, version 3.1. The H&E- and picro-Sirius-stained sections were analyzed under a Leica DMLB 80 microscope connected to a Leica DC300FX camera. After image digitalization, we measured each experimental liver area per group under 20× magnification in five fields. The fibrosis degree analysis was performed using the criteria reported previously [74]. 

### 2.6. Pharmacokinetic Study

To determine the pharmacokinetic profile of the Cit-MnFe_2_O_4_, we determined classical pharmacokinetic parameters that are commonly used, such as $T_{1/2}\;$ (half-life) and the area under the curve (AUC). We adapted the concept of drug exposure to the MNP bioavailability, which was calculated from the area under the liver curves of the two experimental groups. Regarding the liver signals, we quantified the highest MNP level detected ($C_{Max}$) and the time to the highest MNP level ($T_{Max}$).

#### Analysis of the Ex Vivo Biodistribution of the Cit-MnFe_2_O_4_

After the in vivo measurement to collect the quantitative information, the rodents from both groups (SAL and DEN/TAA) were euthanized at 60 min by decapitation after the MNP injection. Subsequently, the organs of interest, such as the liver, spleen, heart, lungs, and kidneys, were collected by a laparoscopy procedure. In this experimental procedure, we also collected a blood sample. To quantify and certify the mass of MNPs accumulated in each organ, we randomly picked a sample (100 mg) of each organ and the blood, which had been previously lyophilized, homogenized, and stored in a volume-controlled flask. According to the previous protocol, the samples were positioned on the ACB sensor for signal detection to determine the mass of MNPs using a calibration curve that was previously constructed from an MNP batch (initially 28 mg/mL) diluted into fourteen vials with different concentrations while controlling the volume. This procedure allowed for the comparison of the measured ACB signals to established MNP concentrations [42]. 

### 2.7. Statistical Analyses

Data were expressed as means ± standard deviations. An unpaired Student’s *t*-test was used to compare the control and the treated groups’ pharmacokinetic parameters (T_1/2_, AUC, and biodistribution quantifications). The incidence data from the histological analysis were analyzed using Fisher’s exact test. The other data were compared using the Mann–Whitney test or Student’s *t*-test, considering a significance level of *p* < 0.05. Analyses were performed using GraphPad Prism 6.0 software (GraphPad, San Diego, CA, USA).

## 3. Results

### 3.1. MNP Characterization

We synthesized manganese ferrite nanoparticles coated with citrate (Cit-MnFe_2_O_4_) through the co-precipitation method. These MNPs were applied due to their excellent field magnetic response to the ACB system. At a concentration of 28 mg/mL, the MNPs showed a superparamagnetic behavior. According to our TEM results, they presented a core diameter of 24 ± 4 nm. Once the organic molecule citrate was small (from 1.5 to 10 nm), it was assumed that the MNP core indicated by the TEM images was equal to the diameter of the Cit-MnFe_2_O_4_ MNPs. Through the DLS Zetasizer results, the MNPs presented with a hydrodynamic size of 65.6 ± 4 nm, a polydispersion index for the colloid sample of 0.25, and a zeta potential of −27.8 mV. We observed a negative zeta potential for the magnetic Cit-MnFe_2_O_4_ (−27.8 ± 1.7 mV), which resulted from their surface being coated with citrate ions due to the effect of citrate adsorption onto bare MNPs. This value is in agreement with literature reports [69,74].

The assessment of magnetic characterization for the powder (pure MNPs) and colloidal solution (magnetic fluid) using an ADE Vibrating Sample Magnetometer (VSM) indicated a magnetization saturation of 52.8 emu/g. The magnetization profile showed a quasi-static superparamagnetic behavior (no coercive field at DC conditions) (see Figure 2C of [69]). We also confirmed the presence of Mn and Fe in the MNPs through an EDS analysis. The XRD analysis showed the structural characterization of the Cit-MnFe_2_O_4_ MNPs. The XRD pattern of as-dried MnFe_2_O_4_ confirmed the ferrite phase’s formation. The diffraction peaks matched the single crystalline MnFe2O4 (JCPDS card No. 074–2403). The XRD results for MnFe_2_O_4_ were comparable with the previously reported results [75]. It is worth pointing out that we did not detect any impurity phases in the ferrite group. See Figure S5, Supplementary Materials. We confirmed the presence of the magnetic core and citrate shell through a Fourier-transform infrared (FTIR) analysis, observing bands at 1581 and 1383 cm^−1^ for the Cit-MnFe_2_O_4_ MNPs (black curve), which were assigned to the citrate due to the C-O bonds of the carboxylic group present in the molecule [76]. The absorption peak within 500–600 cm^−1^ corresponded to the Fe–O vibration, which was related to the magnetic phase [77]. All results of the MNP characterization process are described in the Supplementary Materials.

### 3.2. Macroscopic Aspects of Animals Subjected to Cirrhosis Associated with Hepatocarcinogenesis 

**Figure 2:**
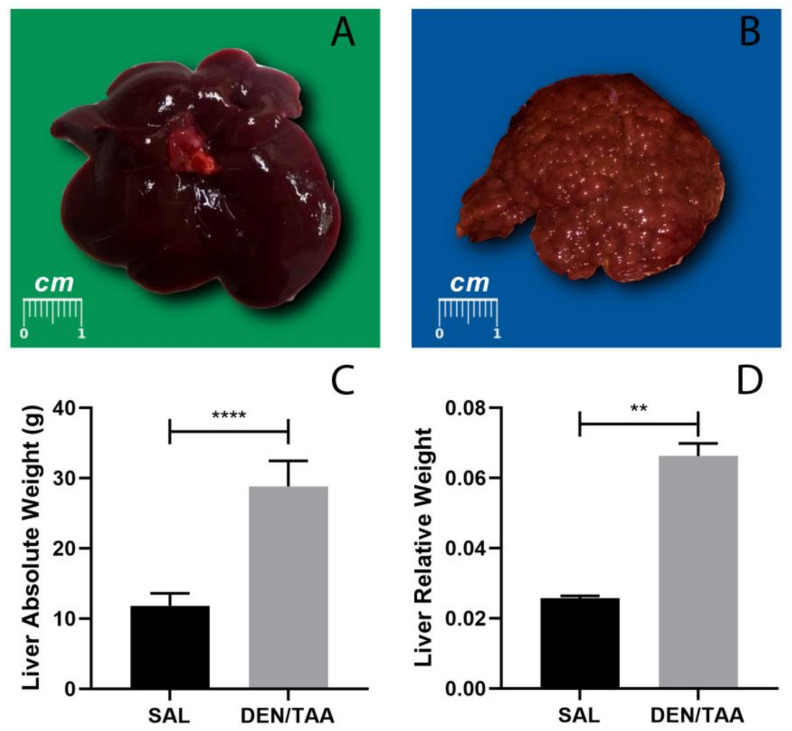
Representative images of macroscopic aspects of livers from (**A**) SAL-group animals and (**B**) DEN/TAA-group animals. Analyses of the absolute liver weight and relative liver weight are shown for (**C**) the SAL and (**D**) the DEN/TAA group. The relative liver weight (LW/BW) is expressed as the ratio between the liver weight (LW) and the body weight (BW). LW/BW ratio values are expressed as means ± sd; for the SAL and DEN/TAA groups, they were 0.25674 ± 0.000706 and 0.066253 ± 0.003554, respectively. (** *p* < 0.05) and (**** *p* < 0.0001).

Macroscopically, the livers from animals of the SAL group (Figure 2A) presented typical features (regular and smooth surfaces). On the other hand, the livers from animals of the DEN/TAA group (Figure 2B) presented rough surfaces with numerous nodules. These findings indicate that the animals subjected to the DEN/TAA protocol presented well-defined features of cirrhosis. In addition, as expected, the DEN/TAA treatment increased the animals’ absolute and relative liver weight (RLW) (Figure 2C,D, respectively).

### 3.3. Histopathological Analysis, Collagen Morphometry, and Immunostaining

**Figure 3:**
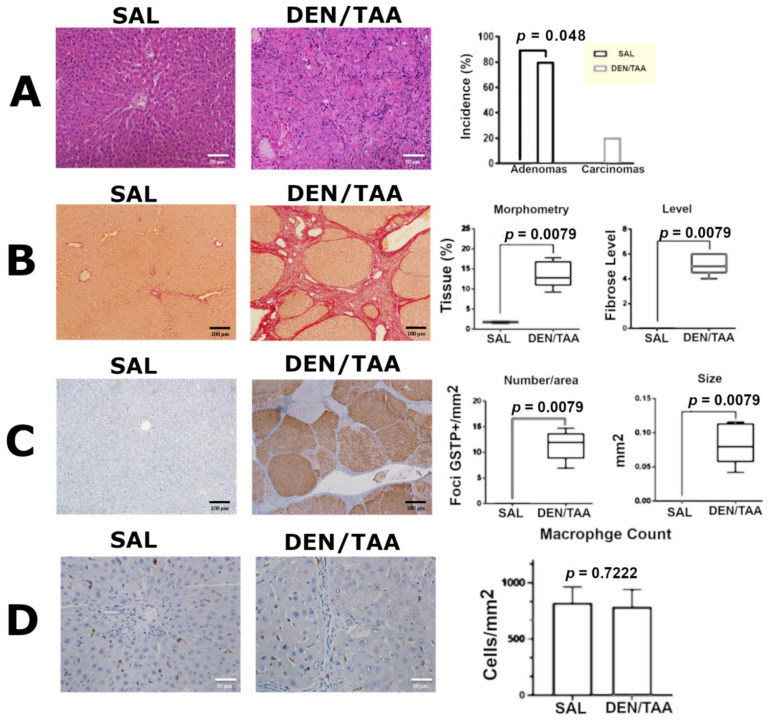
(**A**) Representative images of H&E-stained sections of SAL (left) and DEN/TAA (center) livers; HCC was characterized by profound cellular atypia and was composed of malignant hepatocytes arranged in acinar structures in the DEN/TAA group. Data on the incidence of adenomas and carcinomas are presented in terms of proportion (%) of affected animals and were analyzed by Fisher’s exact test (*p* < 0.05) (right). (**B**) Collagen analysis shown with picro-Sirius red, showing sections from the SAL (left) and DEN/TAA (center) groups. Morphometry and degree of fibrosis data are presented in box plots and were analyzed using the Mann–Whitney test (*p* < 0.05) (right). (**C**) Immunohistochemistry sections from the SAL group (left) and the multiple GST-P+ nodules in the DEN/TAA group (center). The number and size of GST-P+ lesions are presented in box plots and were analyzed using the Mann–Whitney test (*p* < 0.05) (right). (**D**) Immunohistochemistry for the CD68 marker in sections from the SAL (left) and DEN/TAA (center) groups, showing macrophages. Macrophage count data are presented as means and standard deviations and were analyzed using a *t*-test (*p* < 0.05) (right).

The histopathological analysis revealed that 80% (*p* = 0.049) and 20% of animals in the DEN/TAA group developed adenomas and HCC, respectively (Figure 3A). Compared to the SAL (control) group, the livers from the DEN/TAA group presented with multiple preneoplastic lesions and nodules that were positive for GSTP (Figure 3B, *p* = 0.0079). Sirius red-stained DEN/TAA liver sections demonstrated extensive collagen deposition (Figure 3C, *p* = 0.0079) with bridging fibrosis and cirrhotic nodules, and most were positive for GST-P (fibrosis level 5, *p* = 0.0079). Figure 3D shows the data for the immunohistochemical analysis of the CD68 marker. The results indicate that no statistical difference was observed in the macrophage counts between the SAL and DEN/TAA groups (*p* > 0.05).

### 3.4. Dynamic ACB Monitoring

**Figure 4:**
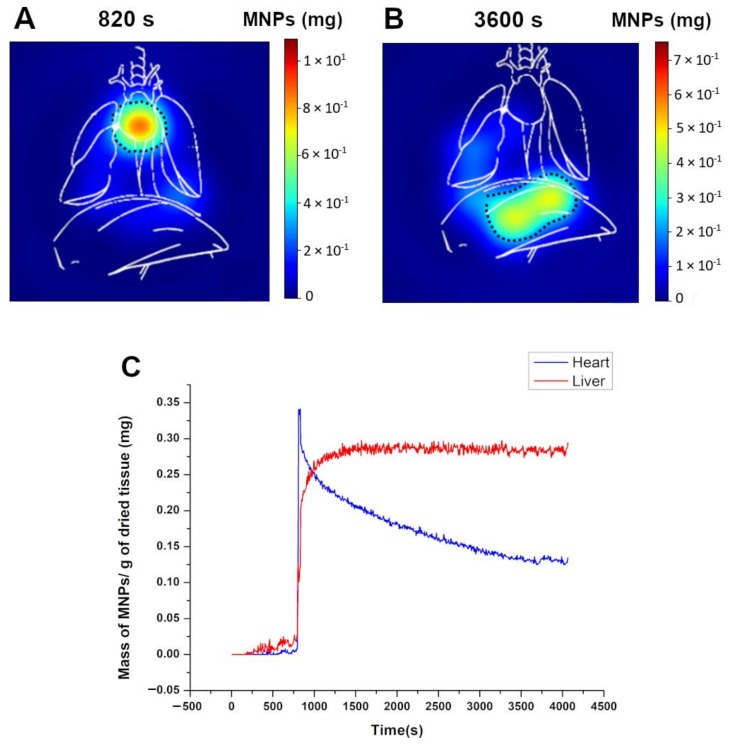
Representation of the biodistribution by frames after injection and the specific ROIs selected to access the pharmacokinetic parameters; (**A**) 820 s, showing the high and low concentrations of MNPs in the heart and the liver, respectively; (**B**) 3600 s, indicating the final biodistribution process, which was characterized by a solely higher intensity signal in the liver; and (**C**) the average intensity over time for ROI 1 (heart region) and ROI 2 (liver).

We acquired images that dynamically represented the biodistribution of MNPs in real time through MC-ACB monitoring. The images were acquired sequentially, allowing for a video representation of the circulation and accumulation processes. Figure 4 presents two frames showing moments of the MNP biodistribution for both experimental groups. In the first frame, at t = 820 s, an ROI was selected in the heart. The second frame, at t = 3600 s, corresponds to the liver region. These ROIs were applied to all the imaging frames, generating biodistribution curves. Previously, we positioned the animals on the MC-ACB system at the same projection to ensure the animal’s anatomical references were kept during the biodistribution acquisition.

Consequently, Figure 4A shows the arrival of MNPs in the heart right after the MNP injection, while Figure 4B shows the final accumulation in the liver region. In order to demonstrate the difference between the MNP distributions, we quantified the average distribution of MNPs in the ROIs of the images. Figure 4C shows the MNPs in the bloodstream and liver. A high-intensity peak characterized the arrival of MNPs in the heart shortly after MNP injection. Then, the distribution of MNPs was represented by a rapid decay in the heart signal. Simultaneously, the liver captured and removed the MNPs from the bloodstream due to the high blood supply and many Kupffer cells in the hepatic tissue.

The liver signal can be associated with the uptake of macrophages and the accumulation of MNPs in the parenchyma. As depicted in Figure 4C (red curve), the liver showed a saturation tendency over time after a rapid intensity increase.

### 3.5. Pharmacokinetic Assessment and MNP Biodistribution

The kinetics of MNP accumulation were evaluated to prove the liver performance during MNP uptake. The kinetics of MNP accumulation were assessed by plotting graphs of the liver signals previously obtained from ROI imaging. To determine the liver’s accumulation, we employed the classical concept of the AUC. We found a significant difference (*p* < 0.0001) between the rates of MNP deposition in the hepatic tissue of the animal groups. The pharmacokinetic assessment of hepatic curves also indicated significant differences (*p* < 0.0001) in $C_{max}$ and $T_{max}$ for the DEN/TAA group. The evaluation of liver signals revealed that the healthy livers reached $C_{max}$ after 26.63 min ($T_{max}$).

On the other hand, the livers under a cirrhosis-induced process had an inversed profile, presenting with a lower peak concentration that was reached in a shorter time ($T_{max}\;$ of 16.7 min) and remained constant over time. Through the plotted heart curves, we assessed the $T{\;}_{1/2}\;$ of the MNPs. The exponential decay analyses of the DEN/TAA group showed a half-life of 16.3 min compared to 28.3 min for the SAL group. All the pharmacokinetic parameters of the DEN/TAA group were found to be significantly different from the SAL group (Student *t*-test, *p* < 0.05). The pharmacokinetic parameters are summarized in Table 1.

**Figure 5:**
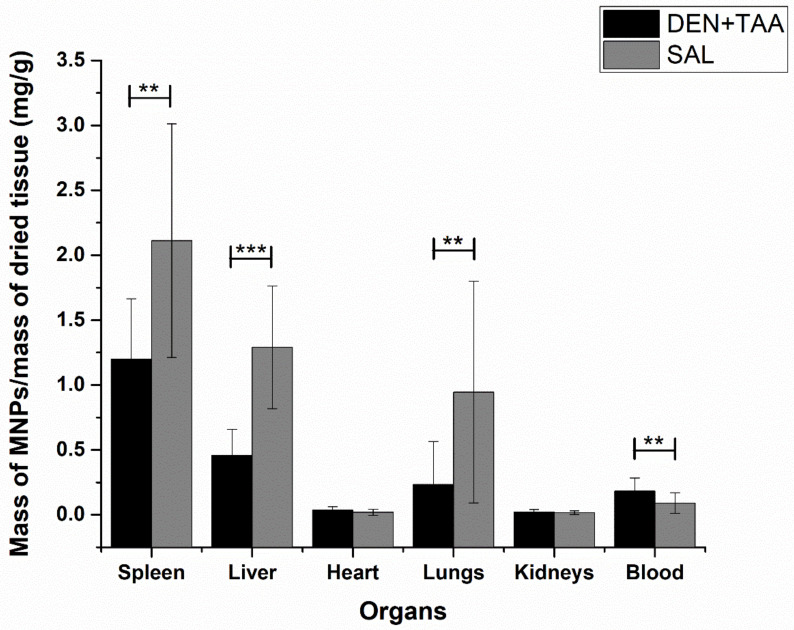
ACB data for MNP biodistribution in SAL and DEN/TAA; ** *p* < 0.05; *** *p* < 0.01.

After acquiring the signals from the in vivo biodistribution measurements and the euthanasia of the animals, we started the protocol to analyze the ex vivo biodistribution from the collected organs as described in Analysis of the Ex Vivo Biodistribution of the Cit-MnFe_2_O_4_ section. From our system’s ACB characterization, we found a limit of detection (LOD) of 12 µg and a limit of quantification (LOQ) of 40 µg for the MNP reference. The sensitivity was 0.9 (χ/mg (MNPs). Figure 5 presents the profile for the ex vivo MNP biodistribution. In general, we noticed a similar behavior of the MNP biodistribution between the experimental groups, where the spleen retained most of the particles, followed by the liver and lungs. However, we found significant differences between the control and treated groups for the same organs (spleen, liver, and lungs), indicating a higher accumulation for the SAL group. The ACB quantification revealed that the MNP accumulation in organs such as the heart and kidneys was minimal, with these organs presenting with very low values of MNP deposition. Both organs do not typically specialize in MNP retention and capture, which would explain the low signal. In addition, the two organs did not show significant differences between the groups, suggesting that besides the MNP properties, which would facilitate the splenic and hepatic uptake, the induced liver injury could not influence MNP deposition in these organs.

The biodistribution analysis also indicated differences in the MNPs found in the blood samples. At the end of 60 min, the amounts of MNPs found in the SAL group’s spleen, liver, and lungs were significantly higher than those in the DEN/TAA group.

## 4. Discussion

Besides its properties such as an excellent low-field magnetic response and a high magnetization saturation, Cit-MnFe_2_O_4_ presents with a negative zeta potential. According to the literature, nanomaterials with positive zeta potentials show an increased clearance [78,79,80,81]. It is generally known that positively charged nanoparticles have faster blood clearance, while neutral and negative particles exhibit a longer circulation time [82,83,84,85]. In addition, nanoparticles with a negative charge present with a lower Kupffer cell uptake in addition to their higher circulation time, which contributes to an increased tumor uptake [86,87]. Furthermore, a strongly negative potential allows the particles to be stable over a variety of pH levels and effectively prevents agglomeration due to steric and electrostatic forces from the citrate layer. The MNPs employed in this study were selected due to their ability to act exclusively as tracers for in vivo measurements, as the aim of this work was to assess liver cirrhosis under the MC-ACB system. Therefore, manganese ferrite nanoparticles are not functionalized with biotherapeutics and chemotherapeutics to work as cirrhosis treatment vehicles based on drug delivery systems and gene therapy.

Although there have been numerous alternatives to the treatment of chronic liver diseases such as cirrhosis and HCC, limitations such as non-specific targeting and adequate drug delivery concentrations have reduced the chances for the successful treatment of these illnesses. Therefore, new agents with improved therapeutic efficiencies have been investigated. However, new translational studies assessing the pathophysiological and pharmacokinetic profiles in liver cirrhosis are essential, as they will contribute to new perspectives in the approach and investigation of new drugs. 

In the present study, we assessed the biodistribution profile of MNPs in a rat cirrhotic microenvironment associated with hepatocarcinogenesis, in which a complete assessment performed by the ACB system—which included real-time monitoring and the quantification of the accumulation of MNPs—was evaluated. It is noteworthy that the Cit-MnFe_2_O_4_ used here exhibited great potential for the diagnosis and study of hepatic diseases such as cirrhosis, as previously reported [33]. In addition, this magnetic particle system can be used as an efficient agent in magnetic hyperthermia due to its unique properties [88]. Thus, our real-time in vivo study was performed through image acquisition or distribution profiling of intravenously administered MNPs. The same system was employed to assess the biodistribution process in normal and injured livers in a DEN/TAA protocol.

The different ACB analyses allowed for a complete pharmacokinetic assessment, confirming that the spleen and the liver are the primary organs responsible for capturing MNPs after intravenous injection. The higher retention of MNPs in the spleen and liver can be attributed to the role of the MPS. According to several reports, most injected MNPs are cleared from the bloodstream by specialized cells, such as the resident macrophages of the liver (Kupffer cells) and spleen (red pulp macrophages) [89,90].

The in vivo results were confirmed by an ex vivo biodistribution analysis (spleen, liver, and lungs), indicating that in the DEN/TAA group, a lower uptake of MNPs occurred compared to that in healthy animals. The spleen plays a significant role in the clearance of MNPs from the bloodstream. Due to its close anatomic proximity to the liver, a communication axis between the liver and spleen (“liver–spleen axis”) is commonly reported [91,92]. Furthermore, in the course of cirrhosis with portal hypertension, the spleen volume undergoes an increase in volume that is proportional to the degree of damage to the liver function [4,93]. However, the mechanisms underlying the splenic function under cirrhosis remain unknown. In a study that addressed liver cirrhosis, the authors found a decreased MNP accumulation in the liver. In contrast, the spleen under cirrhosis showed a higher uptake than in non-cirrhotic animals [94]. Despite the spleen’s phagocytic activity increasing with splenomegaly [95], another study indicated that the hepatic uptake of nanocarriers is affected by liver disease, whereas the splenic uptake was partially affected [96]. In addition, another work also found that patients with liver cirrhosis presented with a decreased liver and spleen uptake of a superparamagnetic contrast agent for magnetic resonance imaging [97].

In this way, we believe that liver cirrhosis possibly induces a decreased uptake of the red zone macrophages in the spleen. It is worth pointing out that the red pulp is constituted by the macrophage population, which retains much of the administered nanoparticles.

Firstly, we also hypothesized that the DEN/TAA-induced protocol could have led to an abrupt depletion of the KCs, which is supported by several studies after toxic hepatic injury and infections [98,99]. However, our immunohistochemistry findings with the CD68 marker indicated no statistical differences in the counts of infiltrated macrophages and resident Kupffer cells between the SAL and DEN/TAA groups. It is worth pointing out that the depletion of KCs is not a mechanism related to all types of liver damage. For example, Kessler et al. [100] performed a DEN treatment to induce severe liver damage and did not find a significant KC loss in the short-term or long-term DEN models. 

Concerning the accumulation by the liver, the IV administration of MNPs can reach the hepatocytes. The hepatocytes represent 70% of total liver cells and are separated from the sinusoids by the space of Disse [101]. As mentioned above, hepatic sinusoids are constituted by endothelial cells that have a fenestrated cytoplasm associated with a discontinuous basal lamina [101]. Through their fenestrae, the LSECs allow absorption and secretion to take place across the narrow space of Disse, creating a unique channel for blood–hepatocyte exchange across sinusoids [102]. In addition to being highly porous, hepatic sinusoids are characterized by the absence of an organized basal lamina in healthy conditions.

Nevertheless, hepatic disturbances and diseases such as fibrosis and cirrhosis induce a capillarization process in the LSECs, which leads to the loss of their fenestrated characteristics [103,104]. A continuous basal lamina characterizes the process of capillarization in hepatic sinusoids, thus avoiding the bidirectional traffic of molecules in the blood and the parenchyma, and vice versa [105,106]. Therefore, this could be considered the main reason for the change in the MNP biodistribution. 

By comparing our data for MNP accumulation in the livers of the DEN/TAA group, the results suggest that the increases in intrahepatic vascular resistance, impaired hepatic sinusoidal circulation, collagen deposition, and portal hypertension, which are associated with cirrhosis [107], could influence the arrival of MNPs to the liver. When we analyzed the data for MNP accumulation in the DEN/TAA and SAL groups, the liver presented with a higher significant *p*-value, followed by the spleen and the lungs. We noticed a lower MNP accumulation in the DEN/TAA group, suggesting a decreased hepatic uptake and consequently more MNPs circulating, which was visualized through the statistical differences in the blood analyses (Figure 5). During the liver cirrhosis process, the hepatic tissue undergoes chronic damage and an inflammatory process, during which a repair process is initiated to regenerate damaged hepatocytes, resulting in scar formation. Therefore, we also assumed that besides the capillarization process, there is a loss of hepatocytes in the fibrosis state by connective tissue scars, which could affect MNP uptake, since an altered obstruction of blood circulation with portal hypertension can occur in this chronic disease [108].

Besides liver disorders, chronic liver disease can result in pulmonary complications. In this way, hepatopulmonary syndrome (HPS) is commonly associated with cirrhosis [109]. HPS is a pulmonary disorder characterized by arterial oxygen desaturation, pulmonary vascular vasodilation, and intrapulmonary shunts. The increased shunting associated with pulmonary vasodilatation is responsible for the imbalance between perfusion and ventilation, causing abnormalities in gas exchange [110,111]. This compromised lung profile could explain the lung biodistribution results for the DEN/TAA group, in which the MNPs did not reach the lung alveoli, and consequently did not remain in the tissue.

The pharmacokinetic assessment results confirmed that the DEN and TAA administration model caused damage that altered the liver architecture. Besides the biodistribution results, we noticed that the livers from the DEN/TAA group presented with a limited uptake efficiency of MNP, as observed by the $C_{max}$. We assumed that the altered basal lamina in hepatic sinusoids did not allow blood extravasation towards hepatocytes. In this way, we hypothesized that the new basal lamina in hepatic sinusoids affected the interaction between the blood and the hepatocytes in the DEN/TAA group. In contrast to the control group, the DEN/TAA protocol resulted in an altered blood flow in hepatocytes, which might have decreased liver uptake. Consequently, the non-extravasation towards hepatocytes resulted in early Kupffer cell saturation, according to our values for $T_{max}$. Despite the fact that hepatocytes are not specialized to retain MNPs, the possible deposition in the hepatocytes might be attributed to the diameter of the Cit-MnFe_2_O_4_ MNPs, which was smaller than the fenestrations of the sinusoids [112]. We noticed that the livers under cirrhosis took less time to exhibit a saturation profile, while the healthy livers continued the exchange between the hepatocytes and the hepatic sinusoidal blood. Therefore, we also assumed that a part of the injected MNPs penetrated the sinusoid and reached the hepatocytes in the SAL group. This condition would explain the significant differences in the observed pharmacokinetic profiles, such as the MNP accumulation.

Since our biodistribution results (Figure 5) indicated a lower hepatic uptake for the DEN/TAA group, it instantly led us to consider a longer circulation time of MNPs for this group. As depicted in Figure 2C,D, the DEN/TAA animals presented with an increased liver weight compared to the SAL group. However, to assess the biodistribution profile, we employed a protocol to calculate the mass of particles per gram of lyophilized tissue. The hepatic uptake of DEN/TAA would be higher due to its mass in a quantification using absolute values. It was evident by the non-normalized $T{\;}_{1/2}\;$ values that the livers under these conditions influenced the circulation time of MNPs.

Although the MC-ACB presented a high temporal resolution for acquiring the biodistribution of MNPs dynamically, but only for the liver and heart, we believe that an improvement mainly in the coil array might lead the system to detect the MNP biodistribution in other target organs. 

This study reported an application of a new and improved MC-ACB system compared to the previous one [48], where the main progress was the acquisition of quantitative in vivo images of the MNP distribution in healthy and neoplastic animals. 

In this context, we believe this methodology is adequate for investigating several organs and their functions, either in normal circumstances or while under dysfunction.

Nevertheless, nanotechnology-based magnetic systems are an alternative strategy to the conventional methods for the investigation of liver diseases. These systems can perform non-invasive imaging assessments to work towards an early diagnosis, which might contribute to the efficient delivery of therapeutics to the liver.

## 5. Conclusions

As highlighted by the presented findings, the pharmacokinetic profile of MNP distribution and accumulation was affected by pathophysiological factors induced by a cirrhosis state. Since the liver monocytes and macrophages remained stable, the differences found in the pharmacokinetic profile of cirrhotic animals strongly indicate that hepatic blood flow is most likely responsible for altering the distribution and accumulation profile of MNPs. Therefore, the feasibility of developing nanotechnology-based delivery platforms needs further investigation to address strategies to improve the interaction of therapeutic agents with injured hepatic tissue.

Through an in vivo and ex vivo information acquisition approach, the ACB system provided the ability to monitor and quantify the MNPs in healthy and cirrhosis conditions, providing the requirements necessary to assist in the diagnosis and therapy of hepatic disorders. By extrapolating the possibilities of evaluation to problems found in the clinical environment, the association of the ACB system with MNPs might offer a methodology with easy access, a low cost, and the absence of ionizing radiation to assess several biologic functions under disorders. Furthermore, it is expected that through instrumental improvements, the MC-ACB system will be enhanced to the level of relevant methodologies such as magnetic particle imaging and magnetorelaxometry.

## Figures and Tables

**Table 1:** Pharmacokinetic parameters of Cit-MnFe_2_O_4_ MNPs after intravenous administration at a 32 mg/kg dose for the SAL and DEN/TAA groups. $C_{max}$ = the highest MNP level detected; $T_{max}$ = the time to the highest MNP level; AUC = the area under the curve; $T_{1/2}$ = half-life. **** *p* < 0.0001.

Pharmacokinetic Parameter	Evaluation (Mean ± SD)	
	SAL	DEN/TAA
$C_{max}$ (mg MNP/dose injected)	0.4870 ± 0.01212	0.4150 ± 0.01621 ****
$T_{max}$ (m)	26.63 ± 0.5145	16.71 ± 1.1 ****
$AUC_{0–60\min}$	1472.6 ± 201	1198.5 ± 152 ****
$T_{1/2}$ (min)	19.6 ± 2.3	11.2 ± 3.1 ****

## Data Availability

Almost all data are presented within the manuscript (figures and tables). The raw data presented in this study are available upon request to the corresponding author.

## Supplementary Materials

The following supporting information can be downloaded at: https://www.mdpi.com/article/10.3390/pharmaceutics14091907/s1, Figure S1: (A) Image of MNPs at 100 nm scale. (B) Image of MNPs at 50 nm scale. Figure S2: Hydrodynamic size obtained by the dynamic light scattering experiment. Figure S3: (A) Magnetization curve of MNPs in a fluid sample and a powder sample (B) acquired by the VSM experiment. Figure S4: EDS quantification of the MNPs composition quantification. (A) MNPs sample used, in which the numbers (1, 2, and 3) represent the studied region. (B) Representative example of EDS signal acquired and its quantification (region 2 of the MNPs image). Figure S5. X-ray diffractogram of Cit-MnFe_2_O_4_ MNPs. Figure S6: FTIR measurements of citrate and the Cit-MnFe_2_O_4_ MNPs.
