# Supplementary material for: Biodistribution Profile of Magnetic Nanoparticles in Cirrhosis-Associated Hepatocarcinogenesis in Rats by AC Biosusceptometry
Source: Pharmaceutics. 2022 Sep 8;14(9):1907. doi: 10.3390/pharmaceutics14091907 (PMC9504370; doi:10.3390/pharmaceutics14091907)
Supplement: Supplementary file 1 [file pharmaceutics-14-01907-s001.zip › pharmaceutics-1836277-supplementary.pdf]

1. **Synthesis and characterization of manganese ferrite nanoparticles**

We inserted 50 mmol $FeCl_3$ and 25 mmol $MnCl_2$ into 500 ml of boiling 2.0 mol/L methylamine solution, remaining in a stirring process. In 100 ml of 3% HCl [w/w] solution, $FeCl_3$ and $MnCl_2$ solutions were dissolved. The mixed solution remained constantly stirring, and after 30 min of the reaction, we magnetically removed the solid from the supernatant. The solid was washed three times with distilled water. Shortly, it was acidified with a 0.5 mol/L HNO_3 solution and magnetically separated from the supernatant

The excess iron nitrate was removed from the solution by magnetic decantation after a thermal treatment for 30 minutes (boiling 0.5 mol/L Fe (NO_3). During the process, the precipitate was washed three times with acetone. We prepare the magnetic fluid by separating the precipitate, followed by peptizing the nanoparticles in an aqueous solution.

Afterward, the sample was treated with sodium citrate under stirring for 30 min under a mass ratio of 1:20 of $Na_3C_6H_5O_7$ to manganese ferrite in 50 ml of water. Then, we magnetically collected the precipitate, and the supernatants were discarded. Finally, the desired amount of water was added to the precipitate, washed three times with acetone. Excess acetone was evaporated to form the magnetic fluid sample.

Through a JEOL model JEM-2100 transmission electron microscope (Tokyo, Japan), operating at 200 kV (2.5 Å resolution), the citrate-coated manganese ferrite nanoparticles (Cit-$MnFe_2O_4$) core diameter distribution was obtained by a lognormal. The results indicated a core diameter of 24 ± 4 nm. Figure S1 presents the transmission electron microscope results.

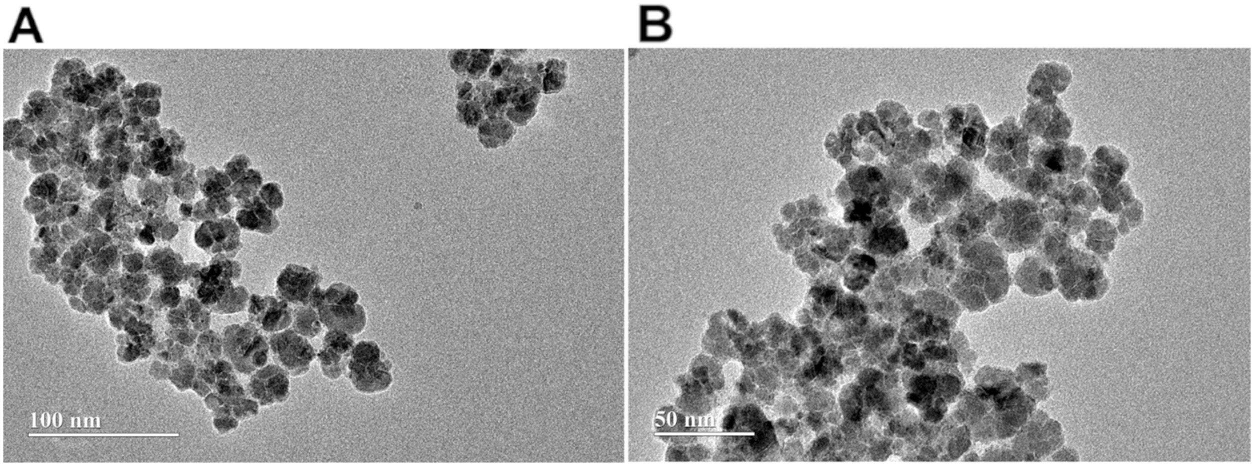

Figure S1. (A) Image of MNPs at 100 nm scale. (B) Image of MNPs at 50 nm scale.

We carried out the dynamic light scattering and Zeta potential experiment through a Zetasizer NanoS (Malvern Instruments, Malvern, U.K.). The hydrodynamic radius (H.D.) was 65.6 ± 4. The sample shows a zeta potential of -27.8 mV at pH 7.4. Figure S2 presents the DLS results.

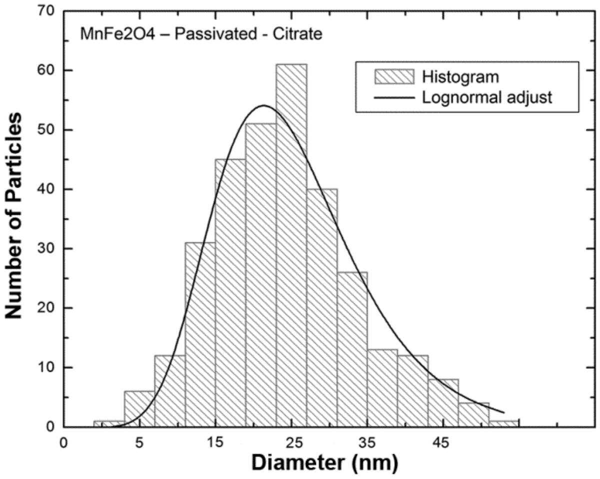

Figure S2. Hydrodynamic size obtained by the dynamic light scattering experiment.

The assessment of magnetic characterization for the powder (pure MNPs) and colloidal solution (magnetic fluid) using an ADE Vibrating Sample Magnetometer (VSM) model EV9 (MicroSense, EastLowell, MA, USA) indicated a saturation of magnetization of 52.8 emu/g (264 emu/cm^3). The magnetization profile showed a quasi-static superparamagnetic behavior (no coercive field at D.C. conditions). Figure S3 presents the magnetization curve of the Cit-$MnFe_2O_4$.

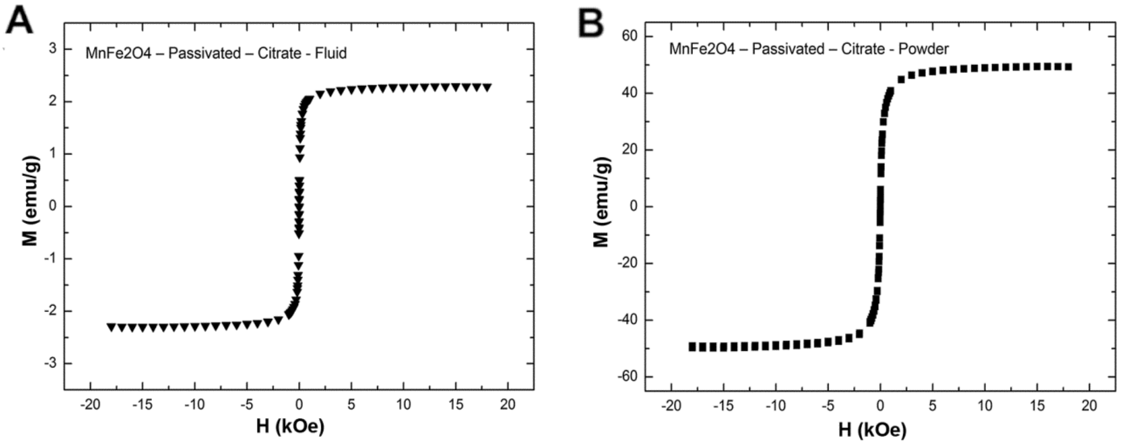

Figure S3. (A) Magnetization curve of MNPs in a fluid sample and a powder sample (B) acquired by the VSM experiment.

We assessed the Cit-$MnFe_2O_4$ composition and formation by Energy-dispersive x-ray spectroscopy (EDS), in which the EDS detector was coupled to the TEM system. We performed fourteen measurements using five MNPs samples. The Iron (Fe) and the Manganese (Mn) content was 74.4 ± 2.6% and 25.6 ± 2.6%, respectively (Figure S4). The Fe: Mn proportion variance is mainly due to the previously described thermal treatment.

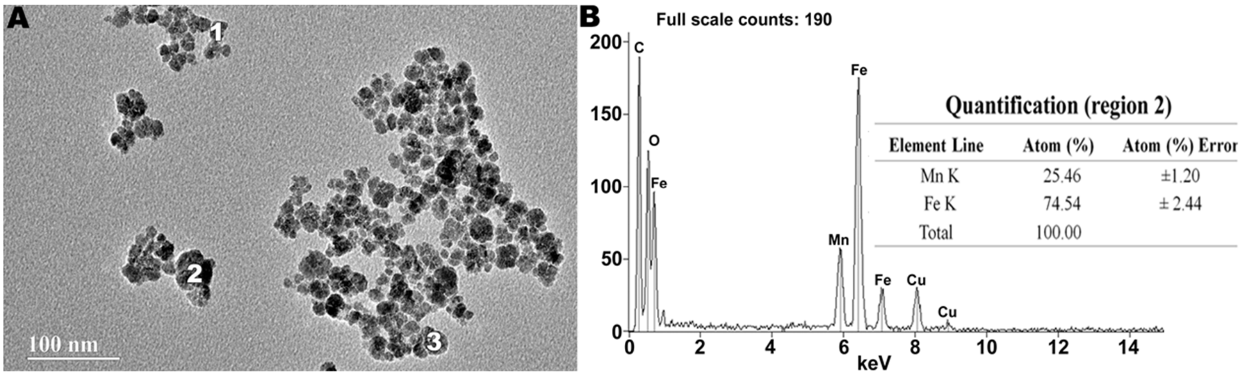

Figure S4. EDS quantification of the MNPs composition quantification. (A) MNPs sample used, in which the numbers (1, 2, and 3) represent the studied region. (B) Representative example of EDS signal acquired and its quantification (region 2 of the MNPs image).

We obtained the X-ray diffraction patterns of the MNPs powders in a Shimadzu 6000 diffractometer (Shimadzu Corporation, Kyoto, Japan) using Cu (Kα) radiation and a 10° to 80° angle range (Figure S5). We employed the Scherrer relation to estimate the crystallite size. The Cit-$MnFe_2O_4$ samples showed a typical structure of spinel-type ferrites.

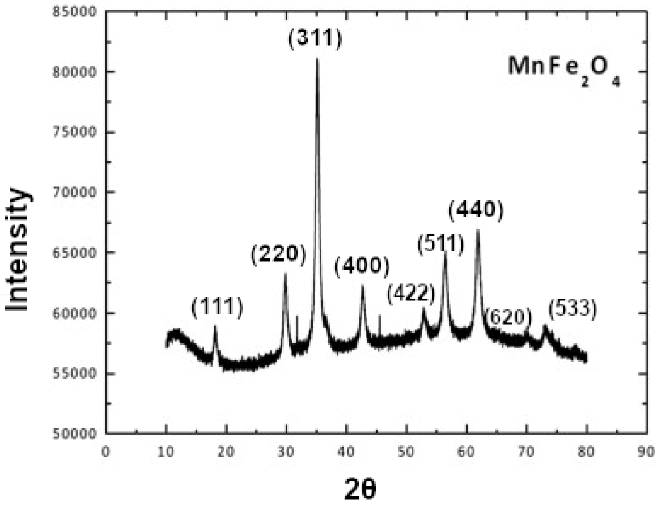

Figure S5. X-ray diffractogram of Cit-$MnFe_2O_4$ MNPs

The XRD analysis carried out showed the structural characterization of the Cit-$MnFe_2O_4$ MNPs. The XRD patterns of as-dried $MnFe_2O_4$ confirm the ferrite phase's formation. Bragg's reflections indexed as (111), (220), (311), (420), (511), (440), (620), and (533) confirmed the constitution of a well-defined single cubic spinel structure. It is worth pointing out that we did not detect the impurity phase along the ferrite group.

We confirmed the presence of the magnetic core and citrate shell through the Fourier transformed infrared (FTIR) analysis, which was carried out using Varian IR 640 equipment.

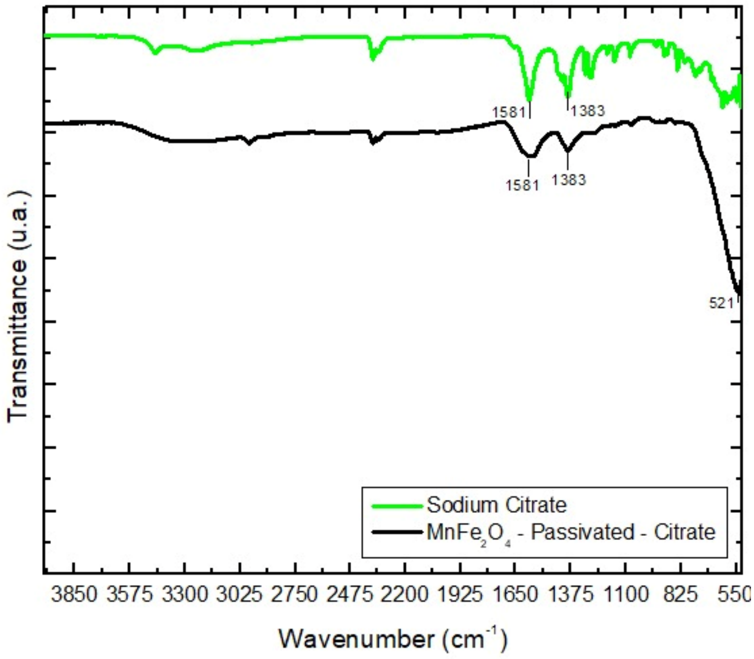

Figure S6. FTIR measurements of citrate and the Cit-$MnFe_2O_4$ MNPs.

We observed bands 1581 and 1383 cm^{-1} present in the Cit-$MnFe_2O_4$ MNPs (Black curve), which is assigned to the citrate, due to the C-O bonds of the carboxylic present in the molecule [1].

Reference

1. Jardim, K.V.; Palomec-Garfias, A.F.; Andrade, B.Y.G.; Chaker, J.A.; Báo, S.N.; Márquez-Beltrán, C.; Moya, S.E.; Parize, A.L.; Sousa, M.H. Novel magneto-responsive nanoplatforms based on MnFe2O4 nanoparticles layer-by-layer functionalized with chitosan and sodium alginate for magnetic controlled release of curcumin. *Materials Science and Engineering: C* **2018**, *92*, 184-195, doi:https://doi.org/10.1016/j.msec.2018.06.039.
